# Study protocol for a randomized double-blinded placebo-controlled trial on ASA therapy for patients with chronic rhinosinusitis with nasal polyps, NSAID-exacerbated respiratory disease, and asthma

**DOI:** 10.3389/falgy.2025.1542481

**Published:** 2025-05-20

**Authors:** Sanna Toppila-Salmi, Annina Lyly, Viljami Salmi, Mikko Nuutinen, Michael Kilpiö, Tanzeela Hanif, Mikko Niemi, Anu Laulajainen-Hongisto, Lena Hafrén, Mika Mäkelä, Paula Kauppi, Paula Virkkula, Alma Helevä

**Affiliations:** ^1^ Department of Otorhinolaryngology, University of Eastern Finland, Joensuu and Kuopio, Finland; ^2^ Department of Otorhinolaryngology, Wellbeing Services County of Pohjois-Savo, Kuopio, Finland; ^3^ Department of Allergology, Inflammation Center, Helsinki University Hospital and University of Helsinki, Helsinki, Finland; ^4^ Department of Otorhinolaryngology—Head and Neck Surgery, Helsinki University Hospital and University of Helsinki, Helsinki, Finland; ^5^ Department of Clinical Pharmacology, Faculty of Medicine, University of Helsinki, Helsinki, Finland; ^6^ Individualized Drug Therapy Research Program, Faculty of Medicine, University of Helsinki, Helsinki, Finland; ^7^ Department of Clinical Pharmacology, HUS Diagnostic Center, Helsinki University Hospital, Helsinki, Finland; ^8^ Department of Pulmonary Diseases and Allergology, Heart and Lung Center, Helsinki University Hospital and University of Helsinki, Helsinki, Finland; ^9^ MD-PhD Programme of the Faculty of Medicine, University of Helsinki, Helsinki, Finland

**Keywords:** chronic rhinosinusitis, nasal polyp, sinusitis, QoL, NSAID-exacerbated respiratory disease (N-ERD), aspirin, acetylsalicylic acid

## Abstract

**Background:**

Chronic rhinosinusitis with nasal polyps (CRSwNP) is a chronic inflammatory condition affecting the nasal passages and paranasal sinuses. It is characterized by persistent inflammation and often leads to a considerable decline in health-related quality of life (HRQoL). A subset of these patients—approximately 17.7%—have NSAID-exacerbated respiratory disease (N-ERD), a more severe form that frequently necessitates repeated sinus surgeries and rescue therapies. Compared with individuals without N-ERD, affected patients are more prone to asthma flare-ups, severe hypersensitivity reactions, and loss of smell. Treatment with acetylsalicylic acid (ASA) following desensitization (ATAD) has been suggested as a therapeutic option in cases of severe CRSwNP with N-ERD. While this approach may offer symptom improvement, decreased polyp burden, and enhanced QoL, it is not without risks, such as gastrointestinal irritation and bleeding complications. This randomized, double-blind, placebo-controlled clinical trial (RDBCT) assesses the effectiveness and safety of ATAD in comparison with placebo in patients suffering from severe CRSwNP, N-ERD, and asthma. The study explores various outcomes, including reduction in polyp burden, improvement in QoL, treatment-related side effects, and biomarker analyses derived from nasal swabs, blood, and urine samples.

**Methods:**

AirGOs Medical is an investigator-initiated RDBCT conducted at Helsinki University Hospital. Participants are randomized to receive either ATAD or placebo. The primary endpoint is the change in the SNOT-22 score observed at the 11-month follow-up. Secondary measures include variations in nasal polyp scores, CRS symptom control, general HRQoL, work productivity loss, peak nasal inspiratory flow (PNIF) with or without acoustic rhinometry (ARM), olfactory function assessed by the Sniffin' Sticks identification test, spirometry, peak expiratory flow (PEF), and histopathological findings at the 12-month follow-up.

**Discussion:**

The AirGOs Medical trial is expected to generate data on the therapeutic value and safety profile of ATAD in patients with coexisting severe CRSwNP, N-ERD, and asthma, potentially informing future clinical practice.

**Trial registration:**

[ClinicalTrials.gov], identifier [NCT03825757]. Registered on 28.2.2019.

## Introduction

Chronic rhinosinusitis (CRS) is defined as persistent inflammation of the nasal cavity and sinuses lasting over 12 weeks, with a reported prevalence of 3%–11% (1, 2). It is divided into two subtypes: CRS with nasal polyps (CRSwNP) and without (CRSsNP). CRSwNP affects 0.5%–4.5% of the population and is associated with significant inflammation, diminished quality of life (QoL), and frequent recurrences despite therapy. The disease imposes both health and economic burdens (3, 4).

NSAID-exacerbated respiratory disease (N-ERD) is a chronic eosinophilic disorder affecting individuals with asthma and/or CRSwNP, triggered by NSAIDs such as acetylsalicylic acid (ASA). It is characterized by a triad of asthma, CRS, and NSAID intolerance, often accompanied by nasal polyps (5). Symptoms such as nasal blockage, wheezing, and dyspnea typically occur 30–180 min after NSAID use (6). N-ERD may appear before or alongside asthma or CRS (7). Among adults with asthma, prevalence is 3%–21%, increasing in severe asthma and CRSwNP cases (8). In Finland, N-ERD affects 1.4% of adults and 17.7% of CRSwNP patients (9). These patients often present with more severe upper airway symptoms, higher CT scores, and a greater risk of polyp recurrence than NSAID-tolerant CRSwNP patients (3, 6, 10).

Conventional CRSwNP treatment includes nasal saline rinses and intranasal corticosteroids. Advanced cases may require systemic corticosteroids, antibiotics, sinus surgery, or biologics (3). N-ERD treatment primarily involves NSAID avoidance and education about safe alternatives, including paracetamol and COX-2 inhibitors such as celecoxib and etoricoxib (3, 6, 7). However, avoiding NSAIDs alone does not halt disease progression (11). While low-salicylate diets have been suggested to help, clinical evidence remains limited.

ASA desensitization involves gradual exposure to ASA to induce tolerance maintained by daily dosing (3). It is typically indicated for poorly controlled CRSwNP or concurrent need for ASA due to cardiovascular comorbidities (12). The desensitization protocol takes advantage of a post-dose refractory period lasting 24–72 h, during which symptoms often improve (3, 13). The Scripps Clinic protocol—gradually escalating to 625 mg twice daily—is commonly used and conducted under medical supervision (3, 14).

ATAD with oral ASA has been shown to improve QoL and reduce polyp recurrence in N-ERD patients. It leads to better symptom control, smaller polyps, and decreased need for additional treatments, such as corticosteroids. Nonetheless, it carries side effects including gastrointestinal irritation, bleeding, and abdominal pain, which can hinder compliance (3, 15, 16). Nasal ASA, in contrast, lacks sufficient evidence for clinical efficacy and has not demonstrated benefit in this patient group (16, 17).

With the rise of safer biologics, evaluating the cost–benefit of ASA therapy remains crucial. This randomized, double-blind placebo-controlled trial (RDBCT) aims to assess the efficacy and safety of ATAD in patients with severe CRSwNP, N-ERD, and asthma. The hypothesis is that ATAD improves QoL and reduces polyp size more effectively than placebo, but with a higher risk of adverse effects. Additionally, nasal samples will be analyzed for genome-wide molecular and microbiome markers, while lipid mediators will be quantified from blood and urine using mass spectrometry and chromatography. These data will be assessed using linear mixed models.

## Materials and methods

### Permissions

The study has been approved by the Ethics Committee (AirGOs Medical, HUS/1801/2017) and National Drug Agency (CT 2017-0015070-42, KL/41/2018) and has been registered to ClinicalTrials.gov (NCT03825757).

### Subjects

The target number of participants for the study is 75 adult N-ERD patients with CRSwNP. This target was determined based on a power calculation, assuming a mean posttreatment difference of at least 13 in the sinonasal outcome test-22 (SNOT-22) between the treatment arms. In a previous study, the response within each subject group was normally distributed with a standard deviation of 17. To detect a true difference of 13 between the experimental and control means, we calculated that 42 experimental subjects and 21 control subjects would be needed to reject the null hypothesis that the population means are equal, with a power of 0.8 and a Type 1 error probability of 0.05. An estimated dropout rate of 15% was factored into this calculation, and if the dropout rate were higher, we would expect smaller effect sizes.

Had endoscopic sinus surgery been performed on 50% of the subjects prior to trial medication, the total number of participants required would have been 150. However, conducting the surgery before starting ATAD was not feasible due to the large number of subjects needed, given the limited number of N-ERD patients available for recruitment in the Hospital District of Helsinki and Uusimaa.

### Primary objective

The primary objective of this study is to assess the relative change in a validated quality of life measure, the sinonasal outcome test-22 (SNOT-22), from baseline to 11-month follow-up between the two randomized treatment arms, i.e., (i) 250 mg Primaspan tablet once daily and (ii) placebo tablet once daily, in patients diagnosed with CRSwNP and N-ERD.

### Secondary objectives

1. Assess the relative change in endoscopic nasal polyp score (NPS) between the two treatment groups at 11 months post-randomization.
2. Assess the relative change in olfactory function and nasal patency measurements between the treatment groups.
3. Assess the relative change in asthma-specific health-related quality of life (HRQoL), as measured by the asthma control test (ACT), among participants with asthma.
4. Assess the relative change in lung function parameters and exhaled nitric oxide [fractional exhaled nitric oxide (FeNO)] between the treatment arms.
5. Assess the relative change in Lund–Mackay (LM) scores on sinus CT scans between the two groups.
6. Compare exacerbation rates, need for rescue treatments, work absences, overall costs, and safety outcomes between the treatment groups.
7. Evaluate clinical parameters during ASA desensitization, including vital signs [ECG, forced expiratory volume in 1 s (FEV1), pulse, blood pressure], physical findings, reported symptoms, peak nasal inspiratory flow (PNIF), and adverse effects.
8. Assess the frequency and nature of adverse effects associated with ATAD.
9. Investigate changes in molecular markers and microbiome composition between the treatment groups.
10. Identify biomarkers predictive of ATAD efficacy and safety.

### AirGOs Medical trial design

**Figure 1:**
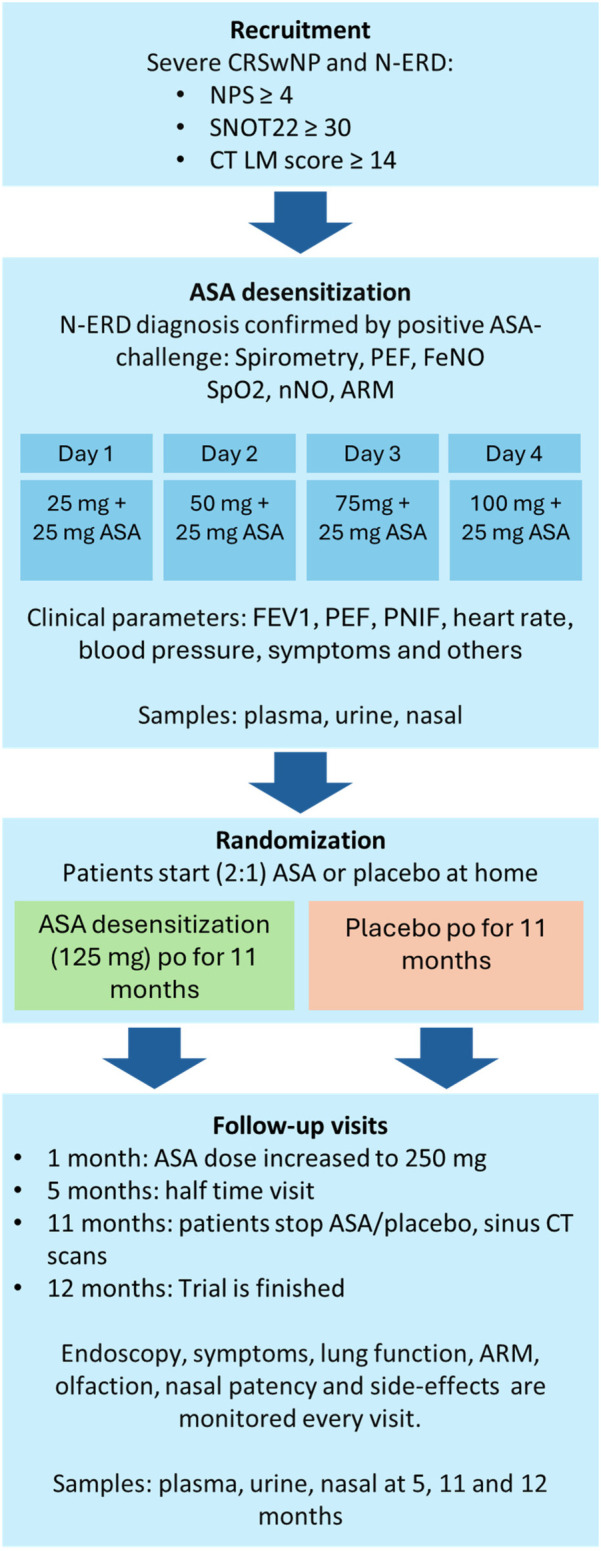
Flow diagram of AirGOs Medical study. CRSwNP, chronic rhinosinusitis with nasal polyps; N-ERD, NSAID-exacerbated respiratory disease; NPS, endoscopic nasal polyp score; SNOT-22, sinonasal outcome test-22; CT, computed tomography; LM, Lund–Mackay score; FeNO, exhaled nitric oxide; SpO2, oxygen saturation; nNO, nasal nitric oxide; ARM, acoustic rhinometry; FEV1, forced expiratory volume in 1 s; PEF, peak expiratory flow; PNIF, peak nasal inspiratory flow.

AirGOs Medical is a randomized, double-blind, placebo-controlled clinical trial. The study is investigator-initiated and was conducted at the Department of Allergology, Inflammation Center, Helsinki University Hospital. Patient recruitment began in 2019 and was completed in 2023. The trial received approval from the local research ethics boards (REBs) of all participating sites. A summary of the study design is presented in Figure 1, with each aspect of the trial described in detail below.

### Eligibility criteria

Table 1 and Table 2 show the inclusion and exclusion criteria, respectively.

**Table 1:** Inclusion criteria.^a^

Uncontrolled CRSwNP, defined as high polyp grade (≥4 bilaterally)
High sinonasal outcome test-22 (SNOT-22) score (≥30)
High Lund–Mackay score of computed tomography scans or sinus cone beam tomography scans (≥14)
Previous ethmoidectomy surgery/surgeries (partial or total) for CRSwNP
A history of at least one of the following:
- >1 oral corticosteroid during the past 2 years - >3 antibiotic courses during the past 2 years This criterion is not required in patients with contraindication/adverse effects during oral steroid use

^a^ Asthma is not an inclusion criterion, although a majority has asthma.

**Table 2:** Exclusion criteria.

Age <18 or >65 years
Prior or current complication of CRS (such as invasive fungal rhinosinusitis or mucocele)
Uncontrolled asthma
Negative result in ASA challenge
Immune system modifying medication or condition - Biologicals/immunosuppressive medication - Immunosuppressive disease/condition [e.g., human immunodeficiency virus (HIV), common variable immunodeficiency (CVID), specific antibody deficiency] - Immunotherapy - Daily use of systemic corticosteroids (prednisolone >10 mg or equivalent)
Use of anticoagulants, selective serotonin reuptake inhibitors (SSRIs) or beta-blockers
A multitude of diseases/conditions - Severe chronic urticaria - Gastric ulcer - Bleeding diathesis - Cystic fibrosis - Primary ciliary dyskinesia - Sarcoidosis - Granulomatosis with polyangiitis - Eosinophilic granulomatosis with polyangiitis - Other severe disease
Communication problems
Unlikely to comply
Pregnancy/breastfeeding

All patients must meet the diagnostic criteria for chronic rhinosinusitis as defined by the 2012 European Position Paper on Rhinosinusitis and Nasal Polyps (EPOS) guidelines (18). Continuous treatment with fluticasone propionate nasal drops and nasal irrigation is required for a minimum of 3 months prior to the first study visit and must be maintained throughout the trial. This treatment regimen is documented at each study visit. If an alternative treatment (e.g., intranasal corticosteroid spray) is used, the reason for its use must also be recorded at every visit.

Patients who are excluded from the study will have the reason for exclusion documented. To reduce selection bias, each study center will systematically screen all CRSwNP patients and record all screening failures. Eligible patients will receive both oral and written information about the study. For those who decline participation, age and gender data will be collected to evaluate the generalizability of the study population.

### Randomization

Patients who meet the eligibility criteria are randomized into two treatment arms in a 2:1 (ASA–placebo) ratio. The active (ASA) and placebo tablets were manufactured by Galena Pharma (Kuopio, Finland), a certified pharmaceutical company specializing in clinical trial products. Galena Pharma is responsible for both supplying the study medications and generating the randomization sequence. The tablets are packaged in sequentially numbered containers according to the randomization list.

The randomization codes are securely stored in sealed envelopes. These envelopes are only opened after the trial monitor has completed the study closure visit and has given explicit permission to open the blind.

### Follow-up

Patients are followed up at 1, 5, 11, and 12 months after randomization. A detailed overview of the follow-up procedures and assessments is presented in Figure 1.

### Definition of CRSwNP and comorbidities

The CRSwNP phenotype is defined according to the EPOS 2020 criteria, characterized by a history of nasal polyps and endoscopic evidence of nasal polyps (3). N-ERD is diagnosed based on a history of typical symptoms (wheezing, nasal obstruction, sneezing, nasal discharge, skin reactions, edema) following NSAID ingestion. Confirmation of N-ERD is made by performing an ASA challenge test in the hospital before randomization (Figure 1).

Clinical data, including history of previous polyp surgeries, time of nasal polyposis diagnosis, allergies, asthma diagnosis, occupation, work environment, smoking habits, medications, and disease history, are recorded by the investigator. Asthma diagnosis is based on a typical clinical history, physical exam, and at least one of the following physiological criteria: (i) recurrent variation of ≥20% in diurnal peak expiratory flow (PEF); (ii) ≥15% increase in PEF with β-agonist; (iii) ≥12% increase in forced expiratory volume in 1 s (FEV1) with β-agonist; (iv) ≥15% decrease in PEF or FEV1 during exercise or moderate to severe bronchial hyperresponsiveness in provocation tests. N-ERD is confirmed by a history of wheezing/cough or naso-ocular symptoms after NSAID use and/or a positive ASA challenge test. Allergy status is confirmed with a positive history and skin prick test or serum allergen-specific IgE against common aeroallergens.

### ASA desensitization before randomization

The ASA challenge and desensitization procedure is conducted for all subjects prior to randomization to confirm the N-ERD diagnosis and assess ASA tolerability. The desensitization process occurs in the daycare department and follows a modified international protocol (19). Given our retrospective data indicating poor ASA tolerability in this population (20), a prolonged desensitization protocol is used. Primaspan (Orion, Espoo, Finland) is employed for ASA desensitization.

The desensitization phase spans 4 days in a hospital setting to closely monitor responses and potential side effects. To confirm the N-ERD diagnosis, patients must demonstrate characteristic symptoms following ASA ingestion, as outlined in Table 3. Symptoms related to the airway, ENT, skin, and general symptoms, as well as FEV1, PEF, and vital signs (blood pressure, heart rate), are carefully monitored.

**Table 3:** ASA challenge positive result criteria.

1	Naso-ocular alone: a 30% or > increase in at least one of the following VAS (0–10 cm) scores: nasal obstruction, nasal discharge, postnasal drip, eye itching. These may exist with or without objective signs of increased nasal turbinate swelling/discharge or eye redness in examination.
2	Naso-ocular (please see 1.) and a 15% or > decline in FEV1 or in PEF (Classic reaction)
3	Lower respiratory reaction only (FEV1 or PEF declines by >20%)
4	Laryngospasm with or without 1–3 (flat or notched inspiratory curve)
5	Systemic reaction: hives, flush, gastric pain, hypotension

On Day 1, patients receive 25 mg + 25 mg ASA, followed by 50 mg + 25 mg on Day 2, 75 mg + 25 mg on Day 3, and 100 mg + 25 mg on Day 4 (Figure 1). ASA is administered at 8 a.m. and 10 a.m. each day. Subjects may take their regular evening medication the night before but must fast from both food and medication on the morning of each ASA desensitization day. Both food and medication can be resumed after ASA administration.

## Results

### Primary and secondary outcomes

The primary outcome is the change in the SNOT-22 score at the 11-month follow-up. Secondary outcomes include changes in the endoscopic nasal polyp score (NPS), LM CT score, HRQoL, loss of productivity, nasal patency [peak nasal inspiratory flow (PNIF) ± acoustic rhinometry (ARM)], olfaction (Sniffin' Sticks identification test), lung function (spirometry, exhaled NO, and PEF), and pathological findings at the 12/24-month follow-up (Figure 1). Safety assessments, including complications and adverse effects, as well as costs and loss of productivity, will be compared between the treatment arms. Additionally, the costs of CRSwNP and asthma treatment 1 year before and 1 year after randomization will be compared by treatment arm. All outcome measurements will be conducted by trained study nurses and investigators. Physiological tests will be performed by a study nurse under the supervision of an investigator and a pulmonologist. Laboratory tests, including skin prick tests, blood tests, and tissue eosinophilia, will be conducted in accredited university hospital laboratories.

### Health-related quality of life (HRQoL)

Disease-specific quality of life is assessed using the SNOT-22, a widely recognized tool that measures the physical, functional, and emotional impacts of CRS. It is a reliable instrument for detecting changes related to interventions (21, 22). The minimal clinically important difference (MCID) for SNOT-22 is 8.9 points for surgically treated patients (21) and 12 points for medically treated patients (23).

General health-related quality of life (HRQoL) is evaluated using two self-administered questionnaires. The EQ-5D-5L assesses five domains: mobility, self-care, usual activities, pain/discomfort, and anxiety/depression (24). The index score ranges from 0 (poor health) to 1 (perfect health), and patients also rate their overall HRQoL using a visual analog scale (VAS), ranging from 0 (worst health) to 100 (best health). The 15D questionnaire, a validated HRQoL tool, measures 15 dimensions of health, including mobility, vision, hearing, breathing, sleep, eating, speech, excretion, daily activities, mental functioning, discomfort, depression, distress, vitality, and sexual activity (25). Respondents select one of five levels for each dimension (1 = best, 5 = worst). The 15D score reflects overall HRQoL, ranging from 0 (dead) to 1 (full health). MCID for the 15D score is ±0.015 (26).

### Olfactory function

Olfaction is assessed using the Sniffin' Sticks identification test, which consists of 12 pens, each containing a distinct common odor. During the test, one pen is placed under the nose for a few seconds at a time. After each presentation, the participant is asked to choose from four options. The forced-choice format is explained to the participant, as it may increase the likelihood of correct responses by chance. The total score is determined by the number of correct identifications out of the 12 odors presented. The MCID for the Sniffin' Sticks test is 5.5 points (27).

### Nasal patency

Nasal patency is assessed using the peak nasal inspiratory flow (PNIF) method with a portable GMI PNIF meter (GM Instruments). A ventilation mask is securely placed over the nose, and the patient is instructed to close their mouth and inhale as forcefully as possible through the nose. The highest inspiratory flow from three maximal inhalations is recorded for analysis. The MCID for PNIF is approximately 20 L per minute (28).

Acoustic rhinometry is used to estimate the cross-sectional diameter of the nasal cavity at various points, with minimal cross-sectional areas recorded. This test is performed after a 15–20 min acclimatization period to allow the patient to adjust to the room's temperature and humidity. A trained nurse or doctor conducts the measurements.

### Lung function

Spirometry is performed at recruitment and at the cessation of medication. It is also conducted if there is suspicion of uncontrolled asthma. The minimal clinically important difference (MCID) for forced expiratory volume in 1 s (FEV_1_) in asthma is considered to be 11% (29).

Mini-spirometry (FEV_1_) is performed on the ASA desensitization days before the first dose, before the second dose, and after the second dose.

Peak expiratory flow (PEF) is measured by patients at home over one week at four timepoints: during recruitment, before ASA desensitization, at discontinuation of medication, and before the end of the trial. The MCID for PEF is an improvement of 12%, as suggested by a study on asthma patients (30).

Fractional exhaled nitric oxide (FeNO) measures airway inflammation, primarily in asthma, reflecting eosinophilic activity and disease control. The MCID for FeNO is a relative change of ≥20% (31).

### Nasal endoscopy

The modified Lund endoscopy score (32) is used to assess polyps, edema, and discharge, with scores ranging from 0 to 2 (33). Polyps are further evaluated using the modified Davos scale, which ranges from 0 to 4 on each side (34):

*Score 0*: no polyps
*Score 1*: small polyps in the middle meatus, not extending below the inferior border of the middle turbinate
*Score 2*: polyps in the middle meatus extending below the inferior border of the middle turbinate
*Score 3*: polyps below the lower border of the middle turbinate, with large medial polyps or any polyp reaching the lower border of the inferior turbinate
*Score 4*: polyps extending below the lower border of the inferior turbinate (35).

The total score (0–8) is calculated by summing the scores for both sides. The MCID for the nasal polyp score (NPS) is 1 point (3, 4).

### CRS disease control

The criteria for assessing clinical control of CRS are outlined in the EPOS guidelines from 2012–2020 (3, 18). Disease control is determined by evaluating common nasal symptoms, sleep disturbances, or fatigue, alongside endoscopic findings and the need for systemic corticosteroids or long-term antibiotics within the past 1 or 3 months (18, 36). Symptom control is defined as a state where patients either have no symptoms or the symptoms are not disruptive. The MCID for overall CRS control has not been universally agreed upon in the guidelines.

### Asthma control

Asthma control is assessed using the asthma control test (ACT) (37) and the Mini Asthma Quality of Life Questionnaire (Mini AQLQ) (38). The ACT evaluates the frequency of shortness of breath, general asthma symptoms, use of rescue medications, the impact of asthma on daily activities, and overall asthma control. Symptoms and activities are rated on a five-point scale, 1 = all the time to 5 = not at all, and for asthma control, 1 = not controlled at all to 5 = completely controlled. Scores range from 5 (poor asthma control) to 25 (complete control), with higher scores indicating better asthma control. A score above 19 suggests well-controlled asthma. The Mini AQLQ consists of 15 questions, covering the same domains as the original AQLQ (symptoms, activities, emotions, and environment). The MCID for the AQLQ is a change of >0.5 (39), and the MCID for the ACT is 3 points (40).

### Exacerbations

Exacerbations of upper or lower airway symptoms requiring increased medication, emergency or outpatient visits, hospitalization, and reduced productivity and sick leave, are documented for the year prior to ASA desensitization and during each follow-up visit over the 12-month follow-up period.

### Productivity, health resource use, and costs

Patients report impaired activity and productivity using the Work Productivity and Activity Impairment Questionnaire (WPAI: GH) (41). Healthcare resource utilization, including medical costs, healthcare services, and productivity costs, is collected from hospital databases, intervention reports, medical records, and national health records. These data are also gathered using the Productivity COST Questionnaire (iPCQ) and the Medical Consumption Questionnaire (iMCQ) (42).

### Adverse effects and safety

Adverse effects related to CRS and asthma treatment in the year prior to enrollment, as well as at each visit and follow-up call during the 12-month period, are documented. Participants are encouraged to contact the investigators whenever needed. Side effects of the investigational medicinal product (IMP) are recorded throughout the follow-up. Any unexpected or sudden adverse effects during the trial are documented in accordance with the 2017 updated Guideline for Good Clinical Practice (GCP) (EMA/CPMP/ICH/135/95). Adherence to the IMP is monitored at each visit through interviews with the study nurse and investigator.

### Tissue and mucus samples

Nasal mucus, epithelial, and polyp tissue samples are collected before randomization and at 5 and 11 months after starting the IMP to examine molecular and cellular changes, as well as microbiome and transcriptome profiles related to efficacy and safety. Participants are also invited to voluntarily donate a blood sample to the Finnish National Biobank for inclusion in a nationwide genome-wide association study (GWAS) project. (https://www.finngen.fi/en).

### Data collection, management, and storage

Clinical data are obtained from electronic patient records, while study information is collected using paper questionnaires. These paper questionnaires, measurement results, and signed informed consent forms are securely stored in locked cabinets. The data are then transferred to electronic case report forms for analysis, stored within a system provided to Helsinki University Hospital by Granitics Ltd., Espoo, Finland.

### Statistical methods

The primary objective of the study is to evaluate the improvement in key endpoints, including the change from baseline to 1 year after starting ATAD, in SNOT-22 scores and the endoscopic nasal polyp (NP) score. Power calculations and randomization procedures for these primary endpoints have been previously outlined. An additional secondary endpoint is the change from baseline in FEV1 at 1 year. The analysis of each imputed complete datapoint will be conducted using an analysis of covariance (ANCOVA) model, linear mixed models, incorporating baseline values, treatment group, prior surgery history, sex, age, and region as covariates. Missing data will be addressed through multiple imputation, and sensitivity analyses will be performed to assess the robustness of the results.

### Monitoring, safety, and reporting of adverse effects

The study is monitored by the Hospital District of Helsinki and Uusimaa's monitoring unit, in conjunction with an independent oversight committee from the Clinical Research Unit at the Academic Medical Center. Monitoring follows International Conference on Harmonization (ICH) guidelines, ensuring the protection of participants' rights and well-being. It also ensures the accuracy and verifiability of data from source documents, and compliance with the study protocol, Good Clinical Practice (GCP), and other relevant regulations. Any serious adverse events are recorded, with those potentially related to the study procedures reported to the principal investigator within 24 h.

### Dissemination

The findings will be published in reputable international scientific journals, and the trial outcomes will be presented at international congresses.

## Discussion

To the authors' knowledge, four randomized double-blind placebo-controlled trials (RDBCT) have previously evaluated the efficacy of oral ATAD in patients with N-ERD and CRSwNP, assessing various outcomes such as symptom control, quality of life (QoL), and airway function (43, 44, 45, 46). Additionally, one randomized double-blind crossover study specifically evaluated ATAD for N-ERD patients (47).

In their RDBCT, Mortazavi et al. (43) administered ATAD (650 mg twice daily for 1 month, followed by 325 mg twice daily for 5 months; *N* = 38 subjects with N-ERD), which resulted in significant improvements in SNOT-22 and symptom scores at 6 months compared with placebo, indicating enhanced quality of life. Furthermore, CT scan Lund–Mackay scores and medication use both decreased. Serum IL-5 levels significantly decreased in the treatment group, while remaining relatively stable in the control group. The same trend was observed with serum IL-4 levels, although the decrease in the treatment group was smaller. FEV1 values improved in the treatment group and slightly decreased in the control group during the study. However, asthma attack rates did not differ between the groups. One patient in the aspirin-treated group experienced gastrointestinal bleeding and withdrew from the study.

Esmaeilzadeh et al. (44) conducted an RDBCT study of ATAD (650 mg ASA twice daily for 1 month and then 325 mg twice daily for 5 months; *N* = 34 subjects with N-ERD and CRSwNP). In this study, FEV1 values significantly increased in the treatment group while slightly decreasing in the placebo group. Improvements were observed in quality of life by SNOT-22, medication requirements, Lund–Mackay score, and symptoms in the treatment group, while the placebo group remained stable. Serum levels of IL-10, INF-γ, and TGF-β did not show significant changes during the ATAD treatment. As for adverse effects, one patient in the ASA group experienced severe intestinal bleeding, and another withdrew due to a skin rash.

Świerczyńska-Krępa et al. (45) conducted an RDBCT study of ATAD (624 mg ASA once daily for 6 months; *N* = 20 subjects with N-ERD and CRSwNP). In this study, ATAD significantly improved asthma control compared with placebo, as assessed by the asthma control test and inhaled corticosteroid dosage. However, no changes were observed in asthma symptoms, FEV1 and PEF values, or the use of rescue medications. ATAD also significantly improved nasal symptoms, SNOT-20 scores, and olfactory function in the treatment group. Additionally, peak nasal inspiratory flow increased, but no changes were observed in CT Lund–Mackay scores or levels of leukotriene E4 or PGD2 metabolites. Four patients in the treatment group dropped out due to dyspepsia.

Fruth et al. (46) conducted an RDBCT study in which ATAD (100 mg ASA daily for 36 months postoperatively; *N* = 70 patients with CRSwNP and N-ERD) improved QoL scores and reduced nasal symptoms significantly in the treatment group. Although non-significant trends indicated that aspirin treatment may improve smell impairment and reduce nasal polyp recurrence, no aspirin-related side effects were reported in this study. In addition to these RDBCT studies with separate placebo and treatment groups, a double-blind crossover study by Stevenson et al. (47) also evaluated ATAD in N-ERD patients. Collectively, these studies suggest that ATAD improves QoL and nasal symptoms and reduces medication use in N-ERD patients. However, there is still some ambiguity regarding how ATAD affects asthma symptoms, as it appears not to prevent asthma attacks, and results across the studies regarding asthma symptoms and FEV1 values are somewhat contradictory. Moreover, side effects such as gastrointestinal symptoms remain a concern, and long-term tolerability needs further investigation. As a result, important questions regarding the safety and efficacy of ATAD remain unresolved.

Targeted biologic therapies have significantly advanced the treatment of Type 2 inflammatory diseases, including N-ERD (48). These therapies are typically considered alongside other advanced options, such as endoscopic sinus surgery and ATAD. The efficacy and safety of biologics for N-ERD patients have been demonstrated in several studies (49). Biologic therapies are recommended for patients who cannot tolerate ASA due to conditions such as gastritis, bleeding risks, or severe uncontrolled asthma (50). However, some patients may require long-term ASA or NSAIDs for other conditions, such as cardiovascular disease, making ASA desensitization a potential option for these individuals (51). To the authors' knowledge, there are no available results from RDBCT studies directly comparing ATAD with biological therapies.

Our randomized double-blind controlled clinical trial assesses the safety and efficacy of ATAD in treating patients with CRSwNP and N-ERD. We will monitor general and disease-specific HRQoL, disease control in CRS and asthma, olfactory function, endoscopic nasal polyp score, lung function, absenteeism, and decreased productivity at work, as well as treatment resource utilization. Adverse events related to the treatments will be monitored throughout the study. Participants first undergo the 4-day desensitization phase and then continue to 11-month maintenance treatment with either aspirin or placebo. Several control visits with doctors during the study will monitor adverse events, disease control, and other factors. After cessation of medication, a posttreatment visit will take place 1 month after stopping the medication. We acknowledge that the study is limited to a single hospital district, which may affect its external validity. Additionally, we recognize that the lack of a direct comparison to biological therapies limits the study's broader impact.

### Trial status

The trial was conducted between 2019 and 2023, and we are currently in the process of analyzing the data.
